# Supplementary material for: Dietary Grape Seed Meal Bioactive Compounds Alleviate Epithelial Dysfunctions and Attenuates Inflammation in Colon of DSS-Treated Piglets
Source: Foods. 2021 Mar 4;10(3):530. doi: 10.3390/foods10030530 (PMC7999447; doi:10.3390/foods10030530)
Supplement: Supplementary file 1 [file foods-10-00530-s001.zip › foods-1091061-supplementary.docx]

**Table S1.** The sequences of primers used for qPCR amplification.

| **Gene** | **Description** | **Accession No.** | **Primer Source** | **Primer Sequence (5′→3′)** | **Orientation** | **Amplicon Length (bp)** | **References** |
| --- | --- | --- | --- | --- | --- | --- | --- |
| *CLDN1* | Claudin 1 | NM_001244539.1 | Pig | CCTACGCTGGTGACAACATTG | Forward | 211 | [1] |
|  |  |  |  | CACTTCATGCCAACAGTGGC | Reverse |  |  |
| *CLDN2* | Claudin 2 | XM_021079578.1 | Pig | CATCCTCTGCTTTTCCTG | Forward | 126 | [2] |
|  |  |  |  | AACTCACTCTTGGCTTTGG | Reverse |  |  |
| *CLDN4* | Claudin 4 | NM_001161637.1 | Pig | CTGCTTTGCTGCAACTGCC | Forward | 106 | [3] |
|  |  |  |  | TCAACGGTAGCACCTTACACGTAGT | Reverse |  |  |
| *CLDN23* | Claudin 23 | NM_001159778.1 | Pig | TGTCTGGCTGAAGGACTCG | Forward | 112 | [4] |
|  |  |  |  | CCACAGGAAAGGAAGGTCAC | Reverse |  |  |
| *CLDN5* | Claudin 5 | NM_001161636.1 | Pig | CCTTCCTGGACCACAACATC | Forward | 110 | [4] |
|  |  |  |  | CACCGAGTCGTACACCTTGC | Reverse |  |  |
| *CLDN14* | Claudin 14 | NM_001161642.1 | Pig | ACGCCTACAAGGACAATCG | Forward | 168 | [4] |
|  |  |  |  | AATGAACTCGGTGTGGGAAC | Reverse |  |  |
| *CLDN20* | Claudin 20 | NM_001159777.1 | Pig | AGCAACATATCCCCATCTCG | Forward | 100 | [5] |
|  |  |  |  | GGCACCTGAAAACTCCATGT | Reverse |  |  |
| *OCCL* | Occludin | NM_001163647.2 | Pig | GAGTACATGGCTGCTGCTGA | Forward | 102 | [4] |
|  |  |  |  | TTTGCTCTTCAACTGCTTGC | Reverse |  |  |
| *ZO-1* | Zonula-1 | XM_003353439.2 | Pig | ACGGCGAAGGTAATTCAGTG | Forward | 111 | [4] |
|  |  |  |  | CTTCTCGGTTTGGTGGTCTG | Reverse |  |  |
| *PTPN2* | Protein Tyrosine Phosphatase Non-Receptor Type 2 | XM_021096020.1 | Pig | ATGGTTTGGCAGCAAAAGAC | Forward | 135 | [5] |
|  |  |  |  | CACACTGAATCCCGTTTCCT | Reverse |  |  |
| *MAGI2* | Membrane-associated guanylate kinase inverted 2 | XM_021102528.1 | Pig | TCCATGGAAAACTCCAAAGC | Forward | 98 | [5] |
|  |  |  |  | AGGGCAGGTTCTGTGGTATG | Reverse |  |  |
| *GNAI2* | G protein subunit alpha i2. | NM_001123116.1 | Pig | TGACAGCATCTGCAACAACA | Forward | 97 | [5] |
|  |  |  |  | GGGGCTGTGTGTGATCTTCT | Reverse |  |  |
| *MYO9B* | Myosin IXB | XM_021083575.1 | Pig | GGCTACAAGGACCTGATGGA | Forward | 94 | [5] |
|  |  |  |  | AGGTGTTGAGGACCAGGTTG | Reverse |  |  |
| *MMP-2* | Matrix metalloproteinase-2 | NM_214192.1 | Pig | GGCTTGTCACGTGGTGTCACT | Forward | 68 | [6] |
|  |  |  |  | ATCCGCGGCGAGATCTTCT | Reverse |  |  |
| *MMP-9* | Matrix metalloproteinase-9 | NM_001038004.1 | Pig | GAAGCTTTAGAGCCGGTTCCA | Forward | 96 | [6] |
|  |  |  |  | GGCAGCTGGCAGAGGAATATC | Reverse |  |  |
| *TIMP-1* | Tissue inhibitor of metalloproteinases 1 | NM_213857.1 | Pig | CAAAACTGCAGGTGGTGATGTG | Forward | 70 | [6] |
|  |  |  |  | CGCAGCCAGGAGTTTCTCAT | Reverse |  |  |
| *TIMP-2* | Tissue inhibitor of metalloproteinases 2 | NM_001145985.1 | Pig | CAGGTACCAGATGGGCTGTGA | Forward | 77 | [6] |
|  |  |  |  | ACTCGTCCGGAGAGGAGATGTAG | Reverse |  |  |
| *CDH1* | Cadherin-1 | NM_001163060.1 | Pig | CAAGTGCCTGCTTTTGTTGA | Forward | 67 | [5] |
|  |  |  |  | AATTTGAGGGCAAACGTGTC | Reverse |  |  |
| *ECM1* | Extracellular Matrix Protein 1 | XM_021089909.1 | Pig | TCTTTTGTCCTGAGGGGTTG | Forward | 65 | [5] |
|  |  |  |  | CTCCCTGACCAGTGAGAAGC | Reverse |  |  |
| *MUC2* | Mucin 2 | XM_021082584.1 | Pig | CCTTGCTCTCGTGTGGAACA | Forward | 191 | [7] |
|  |  |  |  | ACTTCTCCTCGGGCTTGTTG | Reverse |  |  |
| *WNT2B* | Wnt Family Member 2B | XM_003125849.5 | Pig | CCTGAGTTCCTCTCCAGCAC | Forward | 61 | [5] |
|  |  |  |  | AGAGTAAAGCCGCTCCACAA | Reverse |  |  |
| *WNT5B* | Wnt Family Member 5B | XM_021092498.1 | Pig | CACGCCTGGTGTTCACATAC | Forward | 60 | [5] |
|  |  |  |  | GTAGGGCCTGTCTCCTTTCC | Reverse |  |  |
| *FZD3* | Frizzled Class Receptor 3 | XM_001928787.6 | Pig | CTTTGCCTTTGCATGACTGA | Forward | 60 | [5] |
|  |  |  |  | TGCACCTGACTGAAAGCATC | Reverse |  |  |
| *FZD1* | Frizzled Class Receptor 1 | XM_021102361.1 | Pig | TCCCTCCTTTCCCCTTTCTA | Forward | 63 | [5] |
|  |  |  |  | GGAGCCTACCCTCCAGTTTC | Reverse |  |  |
| *TLR-1* | Toll-like receptor 1 | NM_001031775.1 | Pig | TGCTGGATGCTAACGGATGTC | Forward | 102 | [8] |
|  |  |  |  | AAGTGGTTTCAATGTTGTTCAAAGTC | Reverse |  |  |
| *TLR-2* | Toll-like receptor 2 | NM_213761.1 | Pig | TCACTTGTCTAACTTATCATCCTCTTG | Forward | 162 | [8] |
|  |  |  |  | TCAGCGAAGGTGTCATTATTGC | Reverse |  |  |
| *TLR-3* | Toll-like receptor 3 | NM_001097444.1 | Pig | AGTAAATGAATCACCCTGCCTAGCA | Forward | 110 | [8] |
|  |  |  |  | GCCGTTGACAAAACACATAAGGACT | Reverse |  |  |
| *TLR-4* | Toll-like receptor 4 | NM_001113039.1 | Pig | GCCATCGCTGCTAACATCATC | Forward | 108 | [8] |
|  |  |  |  | CTCATACTCAAAGATACACCATCGG | Reverse |  |  |
| *TLR-5* | Toll-like receptor 5 | NM_001123202.1 | Pig | CCTTCCTGCTTCTTTGATGG | Forward | 124 | [8] |
|  |  |  |  | CTGTGACCGTCCTGATGTAG | Reverse |  |  |
| *TLR-6* | Toll-like receptor 6 | NM_213760.1 | Pig | AACCTACTGTCATAAGCCTTCATTC | Forward | 95 | [8] |
|  |  |  |  | GTCTACCACAAATTCACTTTCTTCAG | Reverse |  |  |
| *TLR-7* | Toll-like receptor 7 | NM_001097434.1 | Pig | CCAACAACCGGCTTGATTTAC | Forward | 100 | [9] |
|  |  |  |  | TCTGATTGAAAATAGTGGCTGTTACTACT | Reverse |  |  |
| *TLR-8* | Toll-like receptor 8 | NM_214187.1 | Pig | AAGACCACCACCAACTTAGCC | Forward | 105 | [8] |
|  |  |  |  | GACCCTCAGATTCTCATCCATCC | Reverse |  |  |
| *TLR-9* | Toll-like receptor 9 | NM_213958.1 | Pig | CACGACAGCCGAATAGCAC | Forward | 121 | [8] |
|  |  |  |  | GGGAACAGGGAGCAGAGC | Reverse |  |  |
| *TLR-10* | Toll-like receptor 10 | NM_001030534.1 | Pig | CCTGTCCAACTGCCTCATTTG | Forward | 106 | [8] |
|  |  |  |  | CTAAGTGTTCTAAGGATGTGTTTCTG | Reverse |  |  |
| *MyD88* | Myeloid differentiation primary response 88 | XM_005669362 | Pig | GCAGCTGGAACAGACCAACT | Forward | 66 | [8] |
|  |  |  |  | GTGCCAGGCAGGACATCT | Reverse |  |  |
| *MD-2* | Myeloid Differentiation factor 2 | XM_005663023.1 | Pig | CCTTGTTTTCTTCCATATTTACTG | Forward | 63 | [8] |
|  |  |  |  | CATCAGAGGAATTGCAGATCCA | Reverse |  |  |
| *IRAK1* | Interleukin1 Receptor Associated Kinase1 | XM_003135492.2 | Pig | CAAGGCAGGTCAGGTTTCGT | Forward | 115 | [8] |
|  |  |  |  | TTCGTGGGGCGTGTAGTGT | Reverse |  |  |
| *TRAF-6* | Tumor necrosis factor receptor (TNFR)-associated factor 6 | XM_005652801.1 | Pig | CAAGAGAATACCCAGTCGCACA | Forward | 122 | [8] |
|  |  |  |  | ATCCGAGACAAAGGGGAAGAA | Reverse |  |  |
| *B-2 mg* | β 2 microglobulin | NM_213978 | Pig | TTCTACCTTCTGGTCCACACTGA | Forward | 162 | [10] |
|  |  |  |  | TCATCCAACCCAGATGCA | Reverse |  |  |
| *GAPDH* | Glyceraldehyde-3-Phosphate Dehydrogenase | NM_001206359.1 | Pig | ACTCACTCTTCTACCTTTGATGCT | Forward | 100 | [10] |
|  |  |  |  | TGTTGCTGTAGCCAAATTCA | Reverse |  |  |
| *CypA* | Cyclophilin A | NM_214353.1 | Pig | CCCACCGTCTTCTTCGACAT | Forward | 92 | [10] |
|  |  |  |  | TCTGCTGTCTTTGGAACTTTGTCT | Reverse |  |  |
| *ACTB* | β-actin | NM_213978.1 | Pig | GGACTTCGAGCAGGAGATGG | Forward | 230 | [10] |
|  |  |  |  | GCACCGTGTTTGCGTAGAGG | Reverse |  |  |
| *HPRT-1* | Hypoxanthine phosphoribosyl transferase 1 | NM_001032376.2 | Pig | TGGAAAGAATGTCTTGATTGTTGAAG | Forward | 93 | [11] |
|  |  |  |  | ATCTTTGGATTATGCTGCTTGACC | Reverse |  |  |
| *RPL 32* | Ribosomal Protein L32 | NM_001001636 | Pig | TGCTCTCAGACCCCTTGTGAAG | Forward | 106 | [10] |
|  |  |  |  | TTTCCGCCAGTTCCGCTTA | Reverse |  |  |

1. Luo, X.; Guo, L.; Zhang, J.; Xu, Y.; Gu, W.; Feng, L.; Wang, Y. Tight junction protein occludin is a porcine epidemic diarrhea virus entry factor. *J. Virol.* **2017**, *91*, e00202-17, doi:10.1128/JVI.00202-17 .
2. Zhang, X.-M.; Huang, Y.; Zhang, K.; Qu, L.-H.; Cong, X.; Su, J.-Z.; Wu, L.-L.; Yu, G.-Y.; Zhang, Y. Expression patterns of tight junction proteins in porcine major salivary glands: A comparison study with human and murine glands. *J. Anat.* **2018**, *233*, 167–176, doi:10.1111/joa.12833.
3. Pinton, P.; Braicu, C.; Nougayrede, J.-P.; Laffitte, J.; Taranu, I.; Oswald, I.P. Deoxynivalenol impairs porcine intestinal barrier function and decreases the protein expression of claudin-4 through a mitogen-activated protein kinase-dependent mechanism. *J. Nutr.* **2010**, *140*, 1956–1962, doi:10.3945/jn.110.123919.
4. Pasternak, J.A.; Aiyer, V.I.A.; Hamonic, G.; Beaulieu, A.D.; Columbus, D.A.; Wilson, H.L. Molecular and physiological effects on the small intestine of weaner pigs following feeding with deoxynivalenol-contaminated feed. *Toxins* **2018**, *10*, 40, doi:10.3390/toxins10010040.
5. Rozen, S.; Skaletsky, H. Primer3 on the WWW for general users and for biologist programmers. *Methods Mol. Biol.* **2000**, doi:10.1385/1-59259-192-2:365.
6. Zeni, P.; Doepker, E.; Topphoff, U.S.; Huewel, S.; Tenenbaum, T.; Galla, H.-J. MMPs contribute to TNF-α-induced alteration of the blood-cerebrospinal fluid barrier in vitro. *Am. J. Physiol. Cell Physiol.* **2007**, *293*, doi:10.1152/ajpcell.00470.2006.
7. Quintana-Hayashi, M.P.; Mahu, M.; De Pauw, N.; Boyen, F.; Pasmans, F.; Martel, A.; Premaratne, P.; Fernandez, H.R.; Teymournejad, O.; Maele, L.V.; et al. The Levels of Brachyspira hyodysenteriae binding to porcine colonic mucins differ between individuals, and binding is increased to mucins from infected pigs with De Novo MUC5AC synthesis. *Infect. Immun.* **2015**, *83*, 1610–1619, doi:10.1128/IAI.03073-14.
8. Taranu, I.; Marin, D.E.; Pistol, G.C.; Motiu, M.; Pelinescu, D. Induction of pro-inflammatory gene expression by Escherichia coli and mycotoxin zearalenone contamination and protection by a Lactobacillus mixture in porcine IPEC-1 cells. *Toxicon* **2015**, *97*, 53–63, doi:10.1016/j.toxicon.2015.01.016.
9. Zhang, L.; Liu, J.; Bai, J.; Wang, X.; Li, Y.; Jiang, P. Comparative expression of Toll-like receptors and inflammatory cytokines in pigs infected with different virulent porcine reproductive and respiratory syndrome virus isolates. *Virol. J.* **2013**, *10*, 135, doi:10.1186/1743-422X-10-135.
10. Taranu, I.; Habeanu, M.; Gras, M.A.; Pistol, G.C.; Lefter, N.; Palade, M.; Ropota, M.; Chedea, V.S.; Marin, D.E. Assessment of the effect of grape seed cake inclusion in the diet of healthy fattening-finishing pigs. *J. Anim. Physiol. Anim. Nutr.* **2018**, *102*, e30–e42, doi:10.1111/jpn.12697.
11. Von Der Hardt, K.; Kandler, M.A.; Fink, L.; Schoof, E.; Dötsch, J.; Brandenstein, O.; Bohle, R.M.; Rascher, W. High Frequency Oscillatory Ventilation Suppresses Inflammatory Response in Lung Tissue and Microdissected Alveolar Macrophages in Surfactant Depleted Piglets. *Pediatr. Res.* **2004**, *55*, 339–346, doi:10.1203/01.PDR.0000106802.55721.8A.
